# Limited Evidence of Shared Decision Making for Prostate Cancer Screening in Audio-Recorded Primary Care Visits Among Black Men and their Healthcare Providers

**DOI:** 10.1007/s10903-024-01606-5

**Published:** 2024-06-01

**Authors:** Elizabeth R. Stevens, Jerry Thomas, Natalia Martinez-Lopez, Angela Fagerlin, Shannon Ciprut, Michele Shedlin, Heather T. Gold, Huilin Li, J. Kelly Davis, Ada Campagna, Sandeep Bhat, Rueben Warren, Peter Ubel, Joseph E. Ravenell, Danil V. Makarov

**Affiliations:** 1 Department of Population Health, NYU Grossman School of Medicine, New York, NY USA; 2 Department of Urology, NYU Langone Health, New York, NY USA; 3 VA New York Harbor Healthcare System, New York, NY USA; 4 Department of Population Health Sciences, University of Utah Spencer Eccles School of Medicine, Salt Lake City, UT USA; 5 VA Salt Lake City Informatics Decision-Enhancement and Analytic Sciences (IDEAS) Center for Innovation, Salt Lake City, UT USA; 6 NYU College of Nursing, New York, NY USA; 7 The Fuqua School of Business, Duke University, Durham, NC USA; 8 Sunset Park Health Council, Brooklyn, NY USA; 9 National Center for Bioethics in Research and Health Care, Tuskegee University, Tuskegee, USA

**Keywords:** Shared decision making, Prostate Cancer Screening, Primary Care, Prostate-Specific Antigen test

## Abstract

Prostate-specific antigen (PSA)-based prostate cancer screening is a preference-sensitive decision for which experts recommend a shared decision making (SDM) approach. This study aimed to examine PSA screening SDM in primary care. Methods included qualitative analysis of audio-recorded patient-provider interactions supplemented by quantitative description. Participants included 5 clinic providers and 13 patients who were: (1) 40–69 years old, (2) Black, (3) male, and (4) attending clinic for routine primary care. Main measures were SDM element themes and “observing patient involvement in decision making” (OPTION) scoring. Some discussions addressed advantages, disadvantages, and/or scientific uncertainty of screening, however, few patients received all SDM elements. Nearly all providers recommended screening, however, only 3 patients were directly asked about screening preferences. Few patients were asked about prostate cancer knowledge (2), urological symptoms (3), or family history (6). Most providers discussed disadvantages (80%) and advantages (80%) of PSA screening. Average OPTION score was 25/100 (range 0–67) per provider. Our study found limited SDM during PSA screening consultations. The counseling that did take place utilized components of SDM but inconsistently and incompletely. We must improve SDM for PSA screening for diverse patient populations to promote health equity. This study highlights the need to improve SDM for PSA screening.

## Background

Prostate cancer is the most common non-cutaneous cancer among men in the United States and disproportionately affects Black men; [1, 2] who have the highest prostate cancer incidence and mortality rates [3]. Prostate-specific antigen (PSA) screening is a preference-sensitive decision where harms and benefits can vary significantly across patients. The United States Preventive Services Task Force (USPSTF) Prostate Cancer Screening Statement, as well as the American Urological Association (AUA), the Society of Urologic Oncology (SUO), and the American Cancer Society (ACS) recommend a shared decision making (SDM) approach, where clinicians and patients discuss the evidence and patients are supported to achieve informed decisions [4–6]. 

In spite of changing PSA-screening recommendations, the use of SDM has not yet been widely adopted [7–9]. Men considering prostate cancer screening are rarely encouraged to clarify their preferences or engage in a balanced discussion to weigh the pros and cons of screening [10–12]. USPSTF has recognized the underutilization of SDM in practice, especially among Black men, and has emphasized the need to understand how best to implement and adapt SDM programs within diverse populations. In 2018, the USPSTF changed their recommendation for PSA screening from a grade D (recommending against screening) to C (supporting individualized decision making based on a patient’s balance of benefits and harms) for men 55–69 due to new evidence of long-term benefit and of less harm from overdiagnosis. However, in contrast to the ACS and AUA guidelines [5, 6], which recommend earlier screening of high-risk men, including Black men, the USPSTF makes no recommendations regarding earlier screening of high-risk men [13]. Our team performed an assessment of PSA screening discussions to understand whether SDM was occurring under standard care conditions. As previous research on SDM in PSA screening has relied on self-reported [7, 10–12], we sought to understand these conversations more directly by audio-recording relevant clinic visits. With the overall aim of conducting a qualitative pilot study to guide future intervention development, the objective of this study is to examine the extent of SDM practice in routine primary care appointments.

## Methods

Guided by the indicators of high-quality SDM in the “observing patient involvement in decision making” (OPTION) scoring method [14] and the principles of grounded theory [15], audio-recorded patient-provider interactions were analyzed to appraise the use of SDM in PSA screening decisions. This study was designed to establish a baseline for a larger randomized trial evaluating the efficacy of a Community Health Worker (CHW)-led decision-coaching program [16]. The protocol was approved by the [institution] IRB and written documentation of informed consent was received prior to starting data collection.

### Setting and Participants

The study was performed at an urban Federally Qualified Health Center (FQHC) primary care clinic. Patients were eligible for the study if they were: (1) biologically male, (2) Black, (3) between the ages of 40 and 69 years, and (4) attending the FQHC for routine primary care appointments. Patients were excluded if they were: (1) visiting their provider for any indication other than a well-visit; (2) seen within 9 months of other PSA tests; (3) seen within 180 days after primary diagnosis of urinary obstruction, prostatitis, hematuria, other disorder of prostate, unexplained weight loss, or lumbar back pain; or (4) had a prior diagnosis of prostate cancer (ICD-10-CM C61). The adult male patient population was cared for at the time by 5 full-time primary care providers, all of whom participated in this study. Patient participants were a purposive sample of those who scheduled routine visits during the study period (June through October, 2019).

### Data Collection

Audio-recorded patient-provider encounters were transcribed, and imported into Nvivo software for qualitative data analyses. All patient visits lasted less than 15 min. Study staff set up recording equipment, but were not present in the consultation room.

As discussion around PSA screening was presumed to rarely occur among this patient population, a prompt was used to increase the likelihood that PSA would be discussed. Patients were prompted about PSA in the context of their primary care visit. Providers were handed a document with a prompt to identify the patient as part of the study and to suggest that prostate cancer screening should be discussed in the session. The prompts did not direct the provider or patient as to the content of the PSA discussion.

We determined, a priori, that patients would be sequentially enrolled with ongoing data analysis until thematic saturation was reached [17]. Thematic saturation was quickly achieved (*n* = 13) due to limited PSA conversation leading to no additional learning or new meaningful data collected after this point.

### Data Analysis

Encounter data were coded using procedures designed to ensure thoroughness and reliability. We used Nvivo software to manage the data and coded it according to the principles of ‘Framework for applied policy research’ [18], which consists of a five-stage process including familiarization, identifying themes, indexing, charting, and interpretation. The general development of codes and themes arose from the data, using the principles of grounded theory [15]. To describe the quality of SDM, the OPTION scoring method was applied to each encounter [14]. To enhance objectivity, two researchers took part in the coding and analysis process. All interviews were independently coded by two researchers (AC, KD), who met with another co-author (PU) to resolve discrepancies. When coding was complete, the quotations with each code were examined, summarized, and grouped together into themes.

The OPTION scale is designed to assess the presence/absence and quality of SDM. The OPTION instrument rates a clinician on 12 SDM behaviors. Each item is rated on a skill level of 0 (behavior not observed) to 4 (behavior is exhibited to a very high standard) for a total score range from 0 to 48, with higher scores indicating higher competencies of SDM. The 12 elements of OPTION include: The clinician (1) draws attention to need for decision making process; (2) states there is more than one way to deal with the identified problem; (3) assesses patient’s preferred approach to receiving information; (4) lists options; (5) explains the pros and cons; (6) explores patient expectations; (7) explores the patient’s concerns; (8) checks patient understood; (9) offers explicit opportunities to ask questions; (10) elicits preferred level of involvement in decision making; (11) indicates need for a decision making (or deferring) stage; and (12) indicates need to review the decision. The mean sum OPTION score was transformed to a scale of 0 to 100 using the formula *score*/48 × 100 [14]. Due to sample size, the scores were intended as a descriptor of SDM and not to draw statistical conclusions.

Qualitative codes derived from OPTION explored the discussion of (1) provider recommendations for or against a screening test; (2) patient-specific factors contributing to the need for screening; (3) patient knowledge of prostate cancer; (4) the pros and/or cons of screening; (5) patient preferences; (6) next steps if screening is positive; and (7) final decision outcome.

## Results

### Participants

A total of 16 patients were enrolled with 13 recorded patient-provider encounters, representing 5 unique providers and 13 unique patients. Three patients enrolled in the study were not included in the analysis because two did not complete a clinical visit after enrollment and there was an audio recorder malfunction during one patient-provider encounter. The patients had an average age of 54 (range 40–65) including seven age ≥ 55 years and six age 40–54, with 46% identifying as being married or in a domestic relationship, 54% having only a high school education, and 46% currently employed with pay. The FQHC providers consisted of 2 physicians (providers C, E) and 3 nurse practitioners (providers A, B, D). The providers all identified as female and had an average age of 46 with 4 providers identifying as Black (providers A, C, D, E).

### Shared Decision-Making Quality

A summary of patient-provider PSA screening discussions is presented in Table 1. By the end of the encounter, 11 patients (85%) planned to receive some form of screening (including 1 digital rectal exam) while 2 patients (15%) declined screening. Providers recommended screening to 92% (12) of the patients. The primary reason for the proposed recommendation was based on age (62%) and/or race (69%). Only 3 patients were directly asked about their screening preferences. Few patients were asked about their prostate cancer knowledge (2), potential urological symptoms (3), or family history (6).

**Table 1:** PSA screening interactions between black male patients and their providers during primary care visits at a federally qualified healthcare center

Provider	Patient	Screen Decision*	Provider rec.	Gave Rec. based on…		Discussed…		Asked about…			
				Age	Race	Pros of Screening	Cons of Screening	Screen Pref.	Family History	Symptoms	Prostate Knowledge
A	1	Yes	+	X	X	X	X				
B	2	No	-	X		X	X		X	X	
	3	No	+	X	X	X	X	X	X		
C	4	Yes	+	X	X		X	X			
D	5	Yes	+	X	X	X					
	6	Yes	+		X	X				X	
	7	Yes	+		X	X			X		
	8	Yes	+						X	X	
	9	Yes	+								
	10	Yes	+								X
E	11	Yes	+	X	X	X	X	X	X		X
	12	Yes*	+	X	X	X	X		X		
	13	Yes	+	X	X	X	X				

*Includes a digital rectal screening instead of PSA

Most providers consistently discussed the disadvantages (80% of providers) and advantages (80% of providers) of PSA screening. The most common disadvantages cited by providers were possible false positives (100% of cons discussions and 54% of encounters) and the potential for invasive follow-up procedures (71% of cons discussions and 38% of encounters). The most frequent advantages discussed by providers were ease of screening (67% of pros discussions and 46% of encounters), and the ability to detect cancer earlier (67% of pros discussions and 46% of encounters). On average, the younger 40–54 years old age group had greater SDM discussions with their provider. Clinicians indicated African American men are at higher-risk and that earlier screening is suggested with greater frequency in the 40–54 year-old age group (5/6 participants) compared to the older age group (4/7 participants).

The average patient-level OPTION score was 21.54 (range 0–67); the providers’ average score was 27 (range 1–60) (Table 2). The most frequent, and thorough, noted OPTION elements included when the clinician: (1) draws attention to need for decision making process; (2) states there is more than one way to deal with the identified problem; (4) lists options; (5) explains the pros and cons; and (11) indicates need for a decision making (or deferring) stage.

**Table 2:** Option score elements present per encounter

Provider	Patient	Sum OPTION Score	0-100 OPTION Score	OPTION Element Rating											
				1	2	3	4	5	6	7	8	9	10	11	12
A	1	8	17	1	1	0	0	1	0	0	0	0	0	1	0
B	2	32	67	3	3	0	2	2	0	0	0	2	1	1	1
	3	26	54	3	2	0	0	1	0	1	0	0	0	2	3
C	4	6	13	1	0	0	0	1	0	0	0	0	0	1	0
D	5	4	8	1	0	0	0	0	0	0	0	0	0	1	0
	6	0	0	0	0	0	0	0	0	0	0	0	0	0	0
	7	0	0	0	0	0	0	0	0	0	0	0	0	0	0
	8	0	0	0	0	0	0	0	0	0	0	0	0	0	0
	9	0	0	0	0	0	0	0	0	0	0	0	0	0	0
	10	0	0	0	0	0	0	0	0	0	0	0	0	0	0
E	11	19	40	2	2	0	2	2	0	0	0	0	0	1	0
	12	16	33	2	2	0	1	1	0	0	0	1	0	1	0
	13	23	48	3	3	0	2	2	0	0	0	0	0	1	0

*OPTION elements*: The clinician (1) draws attention to need for decision making process; (2) states there is more than one way to deal with the identified problem; (3) assesses patient’s preferred approach to receiving information; (4) lists options; (5) explains the pros and cons; (6) explores patient expectations; (7) explores the patient’s concerns; (8) checks patient understood; (9) offers explicit opportunities to ask questions; (10) elicits preferred level of involvement in decision making; (11) indicates need for a decision making (or deferring) stage; and (12) indicates need to review the decision.

*NOTE*: OPTION Score elements are rated from 0, behavior not observed, to 4, behavior is exhibited to a very high standard.

### Themes

In addition to the quality codes established a priori, two major themes emerged to describe the PSA-screening-focused interactions. Detailed below, these two themes are: providers explicitly highlighting the importance of SDM and providers stymieing in-depth discussion. Additional quotations supporting these themes are summarized in Table 3.

**Table 3:** Thematic quotes from patient-provider encounters

Theme	Quotes	
Stymieing In-Depth Discussion	**1) Provider D to Patient 10**: So, the test is a simple blood test that we want to do initially…Since I am already doing your annual blood, I’ll just include it. There’s no extra prick, it’s the same one test that I am going to do for everything else, okay? **2) Provider D to Patient 6**: So, we’re going to test your prostate today. **3) Provider D to Patient 8**: I have to re-test your sugar because sometimes if you have sugar, it makes you pee frequently and then we’re going to check your prostate, okay? **4) Provider E to Patient 12**: Regardless, like I said, black men are at higher risk so it is recommended that you start screening a little bit earlier now. Now would be the time, especially now that you are coming to the doctor, checking everything out, doing everything up. Okay?… So which one would you prefer? The blood test or the rectal? Or you can even do both honestly.	
Highlighting SDM Importance	**1) Provider B to Patient 2**: So they left it a little bit more up to the patient, but that makes our job a little more tricky because I can’t just say to you the moment you walk in here, do you want PSA or not? Because you’ll be like I don’t know, you tell me…. **Patient**: Definitely. This is something that I would always like to have a conversation with my doctor regarding testing if it’s recommend[ed] **Provider**: Right, that’s where it’s becoming more of a gray area. It used to be at this age order this test, but now it’s if the patient would like. It’s patient preference now….But then the patients often appreciate some guidance from us in terms of figuring out what that preference is **Patient**: Yeah, I would rather listen to what you tell me because you studied humans so you would know more than what ourselves would know. **Provider**: Like I said, if you walk in the door and I say do you want this test or not. More often than not people would just say what you said initially which is “yeah, do everything, test me top to bottom, I want a complete checkup” **2) Provider B to Patient 3**: …doctors do not know who should be screened for prostate cancer. So it’s a frustrating situation for you because you come to us wanting advice and we don’t know the answer…. It’s difficult for us too because we want to be able to give you advice and we don’t know. It’s based on the science and the science is not clear…. So, this whole conversation is basically to decide whether we order the PSA or not. And you don’t have to decide today…. So, it’s just a difficult decision and this is why they made it something that they’ve thrown it back at you. The patient is the one to make this decision. Our job is to answer any questions that you have, to explain as best as we can, and try to help you with that decision. **3**). **Provider E to Patient 11**: So, in the end, the whole point is for you to be knowledgeable enough about it so you can make a decision what to do. **4) Provider E to Patient 12**: …so, what we do for screening mostly is actually we have a discussion and, in the end, we make the decision together **5) Provider E to Patient 13**: Typically, the recommendation is to have this discussion with you as I am and then essentially the choice is up to you whether or not you even want to pursue screening for prostate cancer. And if you do want to pursue, what do you want to do? Whether it’s the blood test or the rectal exam…. It’s up to you to decide.	
Indicators of SDM Quality		
Provider recommendation	**1) Provider B to Patient 2**: You have no symptoms, no family history, you’re relatively young for the spectrum of men that we would even be screening at all or considering screening for prostate cancer. **Patient**: So, you know I actually would refuse the testing then in that case because, honestly, I don’t want to jump from one place to another and worry myself knowing that I feel okay and fine and healthy…. **Provider**: So, chances are even if we put in the test, it will come out totally normal…. So, you want to skip it for now? **6) Provider C to Patient 4**: I would want to screen you. It’s a blood test but I’m wanting to know how comfortable you are because the screening test is not 100%. **7) Provider D to Patient 7**: So, black men have among the highest incidence of prostate cancer in the world so that’s why it’s very imperative that we do this test. **9) Patient 10 to Provider D**: So, I need to follow up with [the urologist] so I can get my PSA? **Provider**: Well, I’m doing that now as part of this screening test. **Patient**: Okay **Provider**: So, we can either play it two ways which is I can refer you and you can go see him, but I’m going to do the PSA anyway… **10) Provider E to Patient 13**: Necessary is a wrong term. In general, we just kind of recommend what we think is the best and so forth. … Prostate cancer is relatively slow growing type of cancer so, the chances of it causing you to die… is much lower than anything else… That being said, if the screening test that we have for cancer, usually I recommend patients to do some form of screening, I don’t force you obviously but I do recommend it whether it’s one or the other. One that’s quick and easy that we can do here or we can do it through the blood work as I described before…	
Patient-specific factors		
Age and/or race	**1) Provider A to Patient 1**: One thing they are trying to do is to encourage young, black men to screen for their prostate. Usually a lot of men don’t like to get screened. **2) Provider B to Patient 2**: … it should be done in African American males, the recommendation is after 45 years of age. So, the results of the test can be completely negative but an elevated prostate screening or so requires additional testing. Just testing, not treatment…. You have no symptoms, no family history, you’re relatively young for the spectrum of men that we would even be screening at all or considering screening for prostate cancer. **3) Provider B to Patient 3**: …in most cases, men should start discussing prostate cancer screening around the age of 50. So, you’re not 50, but we could say you’re around 50. Today’s your birthday, so you turned 49. But we could say you’re around 50….Now there is more prostate cancer …in black men and obviously if a man has a family history, but you don’t….There is a little bit higher of a risk in all black men, whether or you have a family history or not….Since those are risk factors, those are people who might want to start screening younger than age 50….If you were a black man with your brother or father or grandfather with prostate cancer, that would say we should do it. But you don’t have that family history. **4) Provider C to Patient 4**: …That’s recommended for men between 40 and 45, up to about age 70…The recommendation is a little stronger for black men because for some reason, we’re not sure why, black men tend to have a higher risk of developing prostate cancer and also a higher risk of developing a more severe form that progresses faster. **5) Provider D to Patient 5**: And it should be done in African American males, the recommendation is after 45 years of age….As an African American male, you have a higher risk of developing prostate cancer than other people, have you thought about doing a prostate screening test? **6) Provider D to Patient 6**: Jamaican men have some of the highest rates of prostate cancer on earth. **7) Provider D to Patient 7**: So, black men have among the highest incidence of prostate cancer in the world so that’s why it’s very imperative that we do this test. **8) Provider E to Patient 11**: So, as far as prostate cancer screening goes. It’s recommended starting at age 50, all men are screened or at least have a discussion with their doctor to start screening for prostate cancer. Now just like any other cancer screening that we do, if you’re considered at higher risk, we start a little bit early. High risk for prostate would be black men, if you have a family history you’re considered high risk. Then we’d start earlier, usually 45…You’re 46 now and being African American male, you’re considered a higher risk bracket for having prostate cancer. **9) Provider E to Patient 12**: People at higher risk are people who have family history of prostate cancer, especially if it’s a first-degree relative like your father or brother, but also black men are at higher risk of having prostate cancer…In addition to routine blood work, once you are 45 and older, we also talk about prostate cancer screening in men. Standard is 50 and older, we start screening for prostate cancer. **Patient**: 50 and older? **Provider**: Yes, 50 and older but people who are at higher risk we start earlier so we start at 45. **10) Provider E to Patient 13**: But to kind of go over it again, we start screening for prostate cancer typically at age 50 and people who are at high risk we start a little bit earlier. You’re already over 50 so, you already fit that category…Being a black man, it puts you at a higher risk because there is more prevalence of prostate cancer in black men.	
Family history	**1) Provider B to Patient 2**: Now to this issue, anyone in your family with any cancers, including or not including prostate cancer? **2) Provider B to Patient 3**: Now, I know you said the last time that you didn’t know very much about your family’s history in terms of health problems. But no body with cancer that you’re aware of? …. Like men with cancer? Or problems with the prostate? … If you were a black man with your brother or father or grandfather with prostate cancer, that would say we should do it. But you don’t have that family history. **3) Provider D to Patient 7**: Any prostate cancer in the family as far as you know? **4) Provider D to Patient 8**: Does it run in your family? **5) Provider E to Patient 11**: Let’s talk a little bit about prostate cancer screening. You already did this so obviously you know this is what we are going to talk about because you did the previous part already. Starting off first, anybody in your family that had prostate cancer? **6) Provider E to Patient 12**: Anybody in your family with any type of cancer?	
Symptoms	**1) Provider B to Patient 2**: I haven’t had a chance to ask you yet if you had any funny symptoms like difficulty urinating or feeling kind of like dribbling, or feeling like you have to go but then you get there and you can’t, just anything abnormal? **2) Provider D to Patient 6**: And how many times you waking up at night to urinate? **3) Provider D to Patient 8**: Oh okay, so is it hard to stop the/start the stream when you have to go or you just go frequently? **Patient**: Yeah, it depends on how much I drink **Provider**: oh okay. In the night, how many times do you wake up to urinate? **Patient**: I think on average about twice. Sometimes, I go through the night doesn’t get up, but if you work it off in average it might just be about two times **Provider**: When you do go to the bathroom, does it burn?…Does it itch? Nothing?	
Patient knowledge	**1) Provide D to Patient 10**: What do you understand about the prostate screening process? **2) Provider E to Patient 11**: And do you know much about prostate cancer?…That was going to be my next question, do you know where your prostate is?	
Pros and cons of screening		
Positive reasons for getting screened	**1) Provider A to Patient 1**: Exactly, it’s better to know and do something about it. And just find out later on and it’s too late… **2) Provider B to Patient 2**: …this test is very easy. It’s just a blood test that I would either put in the order or not put it in. You would be having blood drawn already for other kinds of tests like to look at your liver function, just the routine stuff. **3) Provider B to Patient 3**: You wouldn’t feel those symptoms until a more advanced stage…The benefit would be to know earlier. The other thing that I wanted to show is this PSA, which is the test that we use. **4) Provider E to Patient 11**: As far as not screening, is there any risk or benefit to that? Obviously if you don’t screen, you’re much more comfortable. You don’t have to go through all these tests, but then you can obviously miss a cancer that’s there if you don’t do the screening. Prostate cancer in general, grows pretty slowly. It’s not something that when you’re diagnosed, that you die the next day kind of thing. It tends to be a slowly progressive type of cancer. Most men honestly who have even been diagnosed with prostate cancer, even if they do nothing will die of other things before the prostate cancer actually kills them. That doesn’t mean there aren’t any other aggressive cases that can happen also. We can’t predict that. **5) Provider E to Patient 12**: The whole issue with prostate cancer is obviously it is a type of cancer. Any type of cancer we can know more about, treat, eliminate would ideally be the best thing to do. **6) Provider E to Patient 13**: The blood test is a very sensitive test. It means it can pick up a lot of things, which is obviously good because the more things you can pick up, the more you look at and anybody who has cancer probably wants it to be diagnosed more likely than not.	
Down sides to screening	**1) Provider A to Patient 1**: But one thing also when your PSA is elevated, sometimes it is not the cancer. Sometimes as you age, the prostate becomes enlarged as well. It’s part of aging. **2) Provider B to Patient 2**: …Here’s the thing about PSA, it is good in some ways but it is almost too good in the sense that it can be false positive, if that makes sense. There are often times when the test will be positive but it’s not because the person has prostate cancer. There are a whole host of other things that can make the PSA come out positive or elevated. The place where it was becoming burdensome was if the number comes back high, how do we interpret that and do we want to send you off for all these other tests or not? **3) Provider B to Patient 3**: Now, the issue with prostate cancer is that the science is not as clear about the benefit versus harm of screening for prostate cancer. The reason being, the test that we use if we do the screening is called PSA - prostate-specific antigen. What it says here - prostate cancer screening, which is the same as with colon cancer, is done in men who have no symptoms of the disease. It is not clear in this case whether getting screened for prostate cancer can extend a man’s life or help him avoid symptoms or problems… So, the drawback, the downside of PSA is that they can sometimes show up positive even when there’s not a cancer. So, this kind of leads you down this path of doing more and more testing. And even when it does discover cancer, it’s often a cancer that is very slow to progress.…It can be increased for other reasons besides cancer. So, that would be what we might think of as a false positive. Any of these reasons here can make the PSA go up even when someone doesn’t have cancer. **4) Provider C to Patient 4**: Sometimes, we get false indications that there’s cancer when there isn’t…Sometimes, it can miss cancer that is there. **5) Provider E to Patient 11**: In the end, as far as those two modalities, the discussion we’re supposed to have is that obviously no test is 100%, but with prostate cancer screening there’s a high risk of what we call false positives where you have a positive test. More common with the prostate blood test. Let’s say it comes back elevated or high, it could be for other reasons, it could be a lab error, or it could be you have a little inflammation of the prostate that is not cancer that can make your test go high. Let’s say that happens, then what would happen next is that you would have to see a specialist. They have to do a biopsy which can be painful and they would have to test. Biopsy in itself is not 100%. Let’s say you have a cancer and it’s in this part of the prostate, but the biopsy only got this area, you can miss the cancer. **6) Provider E to Patient 12**: …what can happen is the test level can be high and that’ll trigger the thought that you might have cancer, but it could be high for other reasons such as inflammation of the prostate which a lot of men get as they get older. So it might be a little confusing if the number is high. If the number is high, again you would need to do a biopsy test. …Some possible issues that could occur with it is over treatment, over testing, where as if the number is high that kind of forces to have to do…You might have to do numerous biopsies over time to monitor over time, which can be painful in itself. Then sometimes you end up treating just because things aren’t sure but if the number keeps going up they kind of have to treat it and you end up getting surgery and needing other treatment for prostate cancer and so forth. **7) Provider E to Patient 13**: The downside of that is that it can pick up things which we call false positives where it’ll have a high number which will make me think “hey, it can well be cancer” and then really isn’t any cancer. And then would have to go down the line of doing biopsy, doing more tests. It’s just a lot more visits so can have complications in itself with the biopsies. It can be painful, etc. It may not necessarily be warranted.	
Patient preferences	**1) Provider B to Patient 3**: So, ask yourself, do I want to know if I have prostate cancer even if the cancer might never do me any harm? If I found out I had prostate cancer, would I want to be treated considering what we are talking about in terms of these risks? Then the thing that makes it tricky is I think it’s difficult to predict which cancer is going to move quickly and which one is going to move slowly….Would you be willing to accept a high risk of side effects coming from treatment like a surgery, for example, in exchange for a small chance of living longer? **2) Provider C to Patient 4**: I would want to screen you. It’s a blood test but I’m wanting to know how comfortable you are because the screening test is not 100%….Sometimes, we get false indications that there’s cancer when there isn’t….Sometimes, it can miss cancer that is there. How comfortable would you feel if there was a cancer there, would you want to know it? Would you want to be able to do something about it/ to act on it? **3) Provider E to Patient 13**: Then, it probably makes more sense to kind of trend [PSA] and follow it. Are you okay with that? **4) Provider E to Patient 11**: So, the next thing would be to discuss screening for prostate cancer, whether or not you want to screen? Then, which way you want to go about it, whether it’s the rectal exam, or the PSA test, or even both? Any thoughts?	
Final decision outcome		
Plan was made to conduct PSA	**1) Provider A to Patient 1**: Sometimes now [PSA screening] is not required, but usually we have to talk to you about it to see if you want to get it done…so is that something you want me to order today? **2) Provider C to Patient 4**: Okay, so you’d feel comfortable with going ahead and doing the blood test for that?…The cancer screening - okay. So we’re going to do that. **3) Provider D to Patient 5**: Would you like to have the blood test today?…Okay, I’ll add that in the blood work. **4) Provide D to Patient 10**: So, we can either play it two ways which is I can refer you and you can go see [the urologist], but I’m going to do the PSA anyway… **5) Provider E to Patient 11**: I guess we could do the blood one first. We’ll give that a try. If it comes back positive, then maybe we can double check with a more thorough. I think to start off, a blood check should be enough.	
Patient decided not to go forward with screening	**1) Provider B to Patient 2**: So chances are even if we put in the test, it will come out totally normal… So you want to skip it for now? **Patient**: Yeah	
Decision deferred to later appointment	**1) Provider B to Patient 3**: Right, I know it’s a complicated scenario. If I had to boil it down… **Patient**: I’ll just think about it and read on it. If I have any questions, I will come back and ask on it.	
Next steps if screening is positive	**1) Provider A to Patient 1**: But most of the time if it’s very high, usually if it’s higher than normal, we’ll refer you to urology for further testing. And it’s up to if you’d want to do biopsy or continue with that. But it would be highly recommended if the PSA comes back elevated so we can refer you out. **2) Provider B to Patient 3**: So, if your PSA level, if we decide to test it, is high, do not panic. It’s possible that it’s high for reasons unrelated to cancer. If it’s only a little high, often the next step is to have it done again. That’s saying how we would interpret the result if we did the PSA. As of now, we haven’t even ordered the PSA. **3) Provider D to Patient 5**: And it should be done in African American males, the recommendation is after 45 years of age. So the results of the test can be completely negative but an elevated prostate screening or so requires additional testing. Just testing, not treatment…[If the PSA is positive] you will be referred to a specialist and you will be explained all your treatment options. **4) Provider D to Patient 6**: But whatever the result is, there is no need to rush into any decision. It doesn’t grow in one night, and you don’t need to make a decision in one day. **5) Provider D to Patient 7**: The result of this could have multiple implications but whatever happens I don’t want you to rush and do anything. So, we do a test and if the PSA is elevated, then you will go to a specialist, a urologist, but it’s not like it was before. You don’t do it and automatically get a biopsy of it because a biopsy has potential side effects including impotence and incontinence so we don’t do that right away anymore….Unless you have a very strong family history or fast growing malignancy, it’s still just a wait and see. **6) Provider E to Patient 11**: …Let’s say that happens, then what would happen next is that you would have to see a specialist. They have to do a biopsy which can be painful and they would have to test. Biopsy in itself is not 100%. **8) Provider E to Patient 12**: Okay, so we can do your blood test with the rest of your labs. Obviously, if it does come back elevated, then we’ll also have to do a rectal exam and you’ll have to see a specialist who also does a rectal exam and does additional tests, including possibly a biopsy….If the number is high, again you would need to do a biopsy test. Okay?	

#### Highlighting SDM Importance

A theme emerged among providers (40% of encounters) to not only perform SDM but to explicitly mention the importance of having SDM in PSA screening decisions. Providers emphasized the preference sensitive nature of PSA screening telling patients, “it’s becoming more of a gray area. It used to be at this age order this test, but now it’s …patient preference.” Providers described the premise of SDM to explain, “*[patients and providers]* have a discussion and in the end, *[patients and providers]* make the decision together.” Further clarifying that a provider “…can’t just say to you the moment you walk in here, do you want PSA or not? Because you’ll be like, ‘I don’t know, you tell me,’” and explaining “…the whole point *[of SDM]* is for you to be knowledgeable enough about *[PSA screening]* so you can make a decision about what to do.”

#### Stymieing In-Depth Discussion

A second theme emerged showing that when opportunities for SDM arose during patient-provider interactions, providers stymied the potential of in-depth discussion when responding to patient inquiries by providing brief responses and declarative statements that closed the interaction to potential SDM discussion. Many providers limited discussion by using declarative statements with only a confirming follow-up question of “…okay?” at the end (e.g. “I am going to include a PSA test, okay?”). These close ended questions from providers were not followed by inquiries or meaningful feedback from patients. Even direct inquiries by patients regarding PSA screening were sometimes met with a brief uninformative response. One provider, when told by a patient that they, “…thought about *[prostate screening]* but would like to know what the test is all about.” The provider told the patient, “the test is a simple blood test…. Since I am already doing your annual blood, I’ll just include it.”

## Discussion

In our study, the first of its kind to use audio recorded patient-provider conversations to assess the presence and quality of SDM in discussions regarding PSA screening, we found that providers were inconsistent in their counseling approach. While some discussions addressed the advantages, disadvantages, and/or scientific uncertainty of screening, few patients received all elements for SDM. Furthermore, while nearly all providers recommended PSA screening, and most patients received it, very few patients were asked directly about their preferences or their knowledge about PSA screening. OPTION scores were low on average with only 1 of the 5 providers receiving an average score of ≥ 50 (a score of 50 indicates SDM “behaviors are observed and a minimum skill level achieved”). These demonstrated limitations in SDM are consistent with existing literature showing that few men are given the opportunity for SDM during PSA screening [11], and men often do not receive all the information they need to participate in SDM [19, 20]. Our study is an important contribution to and advance over previous work because it employs direct audio recording of the patient-provider interaction, which has similarly been used to evaluate other cancer screening SDM [21], and thus affords some qualitative understanding of these shortcomings.

Our study revealed that, even under ideal circumstances where providers and patients are prompted to discuss PSA screening, there are potential factors influencing the use of SDM beyond the oft-cited barriers to SDM (e.g. limited provider time and belief that SDM won’t influence behavior, patient limited medical literacy) [22]. Specifically, there may be a misunderstanding regarding what constitutes SDM, even among providers who explicitly tell their patients of the need for SDM. A majority of providers stating advantages, disadvantages, and recommendations primarily did so using declarative statements with a confirming question “…okay?” tacked on to the end. This format did not create an opportunity for patients to engage in the conversation or express personal preferences; indeed, no patient in our data set went on to ask any clarifying questions after such a statement. This effect on conversation may also be the consequence of the time constraints of the encounter.

Through the prompting of both providers and patients to discuss PSA screening, participants were likely biased towards potential PSA SDM, as compared to standard care. In spite of this, very limited SDM was noted. Consistent with the literature [10–12], this indicates that, even under the best possible circumstances, where both patient and provider are primed to discuss PSA screening, high quality SDM rarely occurs. Thus, suggesting further efforts are needed beyond clinical reminders to effectively encourage the use of SDM for PSA screening.

Given the persistently low rates of SDM demonstrated in this study and in national trends [20], investigating alternate strategies to promote SDM is crucial. In the context of the indisputably limited time available for counseling [22, 23], an intervention such as decision aids or a CHW/other trained staff -led decision coaching program may be a more feasible alternative [24, 25]. In preliminary work, using an American Cancer Society (ACS) decision aid [6] in a community-based setting, we found a CHW to be potentially as effective as a physician for increasing prostate cancer knowledge and decreasing decisional conflict [6, 26, 27]. A CHW-led approach may address barriers to provider-led SDM by providing more time for patients to receive counseling necessary for decision making, as well as overcome patient-level barriers such as language differences and limited medical literacy [22, 23, 28]. In the context of the larger trial of a CHW-led decision-coaching program [16], these results helped establish a baseline of current levels of PSA SDM and provided additional insights on clinic work flow that informed the decision to implement the ACS decision-aid guided CHW PSA SDM coaching session prior to the appointment [16]. This study had several limitations. Foremost is the limited sample size prescribed by our study design prevents the ability to test deductive hypotheses for both patients and providers and perform subgroup analyses. Furthermore, information was not available on the background of the patient-provider relationships or previous PSA discussions. Therefore, elements of SDM that might have occurred at previous encounters could not be captured. Similarly, by focusing on only visits with Black men, we cannot know if clinician behavior would differ based on patient race. Our study has several strengths including the use of recorded patient-provider encounters to review the exact words and nuanced communication rather than relying on self-report, thus reducing the potential for recall and social desirability bias, and allowing for an understanding of the breadth and depth of such discussions. Third-party observation has a high correlation with patient report, however, observers may perceive fewer SDM behaviors than patients; potentially biasing the audio-recording review towards less SDM [29]. 

## Conclusion

SDM is critical to ensuring the quality of PSA screening, a highly preference sensitive process. Our study found limited SDM during PSA screening consultations among Black men and their providers. The counseling that did take place used components of SDM but only inconsistently and incompletely. Efforts are needed to develop strategies to improve SDM for prostate cancer screening in diverse patient populations which will ensure the quality of PSA screening and promote health equity in a population especially vulnerable to morbidity from prostate cancer.

### New Contribution to the Literature

This research contributes to the current literature by providing evidence, via audiotaped discussions, confirming previous findings that, during PSA counseling, providers may use components of SDM but inconsistently and incompletely. This study highlights the need to further improve SDM for PSA screening.
